# Depression and Cognitive Impairment in a Spanish Sample of Psychoactive Substance Users Receiving Mental Health Care

**DOI:** 10.3390/healthcare10050887

**Published:** 2022-05-11

**Authors:** Bárbara Luque, Victoriana García, Carmen Tabernero

**Affiliations:** 1 Department of Psychology, University of Cordoba, 14004 Cordoba, Spain; ed1gargv@uco.es; 2 Maimonides Biomedical Research Institute of Cordoba (IMIBIC), 14004 Cordoba, Spain; 3 Department of Social Psychology and Anthropology, Instituto de Neurociencias de Castilla y León (INCYL), Campus of the University of Salamanca-Miguel de Unamuno, 37007 Salamanca, Spain

**Keywords:** cognitive impairment, depression, psychoactive substances, alcohol, cocaine

## Abstract

(1) Background: Numerous studies state that the abuse of psychoactive substances produces cognitive, emotional and behavioral disorders. The aim of this study is to analyze the relationship between the consumption of different psychoactive substances with cognitive performance and depression. (2) Methods: The sample was composed of 254 individuals (*M* = 41.81; SD = 10.74, from 18 to 69; 76% male) who received psychological treatment related to the use of substances. Participants were classified according to the main substance consumed: alcohol (42.9%), cannabis (20.5%), cocaine (15.4%), heroin (13%) and benzodiazepines (8.3%). The Montreal Cognitive Assessment and the Beck’s Depression Inventory were administrated. (3) The results indicated no statistically significant differences between levels of depression depending on the substance consumed. Regarding cognitive impairment, it was found that cocaine consumers have the worst level of cognitive impairment, while cannabis consumers have the best level of cognitive functioning. Finally, it was found that participants with severe depression have higher cognitive impairment than those who were diagnosed with moderate depression. (4) Conclusions: Given the high prevalence of depression and cognitive impairment with the abuse of psychoactive substances, early treatment is recommended to avoid a higher cognitive and emotional affectation.

## 1. Introduction

Drug use is a social and public health problem that has worried most countries for decades [1]. The European Monitoring Center for Drugs and Drug Addiction [2] shows that 26.3% of Europeans have used cannabis, 5.2% cocaine and 3.8% amphetamines. In Spain, a sample of high school students found that at least 78.9% have used alcohol at some time, 29.1% cannabis, 3.5% cocaine and 0.6% heroin, which is alarming [3]. There are also acute problems, such as substance overdose with a mortality rate in Europe in 2015 estimated at 20.3 cases per one million inhabitants aged 15 to 64 [3]. According to the latest available data, a rate of more than 40 deaths per one million inhabitants was reported in eight countries in northern Europe, with the highest rates corresponding to Estonia (103 per million) [3].

The consumption of psychoactive substances has multiple adverse effects producing cognitive, emotional and behavioral disorders leading to difficulty in the assimilation of treatments and the acquisition and implementation of new ideas and skills [4]. Drug abuse carries, among other consequences, cognitive deterioration [5] and depression [6]. In relation to cognitive damage, studies developed by Copersino et al. [7] and Bruijnen et al. [8] affirm that the Montreal Cognitive Assessment MoCA [9] is a fast and accurate detection instrument for patients with substance abuse disorder and neuropsychological impairment. Moreover, in another study conducted by Damian et al. [10], it was found that the MoCA was superior in both sensitivity and specificity to the Mini-Mental Mood Examination to assess mild cognitive impairment. So far, there are no studies that have taken into account cognitive impairment in users of different types of drug use, including benzodiazepines. In Spain, 13.5% of the adult population is prescribed treatment with benzodiazepines (14.7% for women and 7.3% among men).

Regarding the level of cognitive deterioration experienced by people consuming psychoactive substances [11], it was found that only 30% of substance users in the study sample had a cognitive performance that could be considered normal compared to the general population, showing that some addicts have cognitive performances that are closer to those seen in individuals with dementia. Regarding the existence of different levels of cognitive deterioration among the different substances consumed, almost none were found, highlighting that heroin users show the worst cognitive functioning and cannabis users showed the best performance [11]. According to several studies, the highest deterioration at the cognitive level can vary depending on the substance consumed, e.g., in alcohol, the damage lies in visuospatial skills, executive functioning, attention, language, abstraction and delayed recall, in accordance with Pelletier et al. [12], and in cannabis users, there is a greater deterioration in immediate recall, delayed recall and verbal capacity [13].

Another repercussion of the repeated consumption of psychoactive substances is emotional disorders [14]. In alcohol-dependent populations, various symptoms of depression and anxiety have been found [15,16]. According to several articles, a greater possibility of association between these symptoms and alcoholism can be found [17,18,19]. On the other hand, it has been shown that cannabis abuse increases the risk of experiencing depression and anxiety in the future [20], where several studies that have been conducted have reported a strong relationship between cannabis, anxiety and mood disorders [21,22,23]. With regard to depression and cannabis users, it has been found that this relationship varies with age, where a depressive disorder is stronger in mid-adolescence and weaker in mature adulthood [24].

As for changes in behavior that the consumption of these substances can induce, an exacerbation of personality disorders has been found, such as antisocial behavior. Among patients with opioid dependence, it was observed that 24% had an antisocial behavior disorder and 17% had a borderline personality disorder [25]. In addition, abusing these types of substances can generate problems in different areas of life such as work, family and personal life. Regarding the workplace, drug use causes two major problems: labor absenteeism and workplace accidents [26]. On the family side, the consumption of these substances can affect coexistence by deteriorating the social relationships of the individual with their surroundings, causing situations of abandonment or abuse [26]. Finally, regarding personal life, the intake of these substances aggravates gender violence [27]. Finally, it is necessary to point out that the consumption of alcoholic beverages contributes to a large number of traffic accidents and judicial problems [28]. Although the present study does not focus on the impact of drugs on the social behavior of users, it seems appropriate to highlight the negative consequences on the social adaptation of substance users.

The main objective of this study was to analyze the relationship between the consumption of psychoactive substances (cocaine, cannabis, benzodiazepines, heroin and alcohol) with cognitive performance and depression. The specific objectives were to analyze the relationship between sociodemographic variables and the different psychoactive substances consumed and to identify the association between depression and cognitive deterioration in users of psychoactive substances.

## 2. Materials and Methods

### 2.1. Participants

The sample consisted of 254 individuals aged between 18 and 69 years old (*M* = 41.81; SD = 10.74). The sample received psychological treatment to stop the use of substances in the drug dependency unit of the public health service of Córdoba, Spain. Participants were diagnosed according to ICD 10 (10th edition of the “International Classification of Diseases”), which, in chapter 5, section 2, includes mental and behavioral disorders due to substance use (F10–F19). Of this sample, 76% were men, and 24% were women. Before collecting the data, a report was sent to the ethics committee of the Andalusian Health Service, considering the privacy of the patients’ information. Participants were selected based on accessibility to health services in Córdoba, where there is psychological assistance for people who engage in the problematic consumption of addictive substances; therefore, the sampling was incidental. Participants were abstinent from the first week after their demand for treatment. However, the participants agreed to take part in the study voluntarily and signed an informed consent form in which they accepted that their answers to the questionnaires would be used for research purposes, knowing that their answers would be treated anonymously.

In terms of consumption, the average age of first consumption was 17.75 years (SD = 6.74), with the youngest at 7 years and the oldest at 50 years old. In the sample, 74.8% started drinking before the age of 18 and 25.2% did so after coming of age. The average length of time they had consumed for was 24.06 years (SD = 11.03), with the shortest consumption duration being less than 1 year, and the longest duration 51 years. Participants were classified according to the main substance consumed: alcohol (42.9%), cannabis (20.5%), cocaine (15.4%), heroin (13%) and benzodiazepines (8.3%). They were also classified according to whether they had depression (64.4%) or not (35.6%), subdividing subjects with depression into three types: mild (23.3%), moderate (14.6%) and severe (26.5%). Finally, they were divided depending on whether or not they experienced cognitive deterioration; according to the MoCA inventory, cognitive deterioration was detected in 78.7% of the sample participants, while the remaining 21.3% were classified as being without cognitive deterioration.

### 2.2. Instruments

A sociodemographic questionnaire was carried out on an ad hoc basis, based on a basic information sheet of admission to treatment, which collects information on sex, age, type of substance consumed, age of onset of consumption and time of consumption.

The Montreal Cognitive Assessment, MoCA [9], is a screening test for mild cognitive impairment or early dementia. The scale has been validated in a Spanish sample of patients with and without cognitive impairment and dementia [29]. Scores range from 0 to 30, where scores equal to or higher than 26 indicate normal cognitive performance, and scores below 26 indicate mild cognitive impairment or early dementia. The duration of this test is about 10–12 min.

Beck’s Depression Inventory (BDI-II) Ref. [30] is a self-report test to assess the presence and severity of depression in adults and adolescents over 13 years of age. The scale has been validated in Spanish with a sample of 470 adults selected from the general Spanish population [31]. It contains 21 items, which correspond to the criteria for the diagnosis of depressive disorders described in the DSM-5 (Diagnostic and Statistical Manual of Mental Disorders). In each of the items, the respondent must choose one of the four existing options that identify their mood in the last seven days. The total score determines the level of depression. Scores from 0 to 9 indicate an absence of depression, from 10 to 19 mild depression, from 20 to 29 moderate depression and above 30 indicates severe depression.

### 2.3. Procedure

Participants were selected for their accessibility to three of the health centers in Córdoba, where medical–psychological assistance is provided for people who engage in problematic substance use, two of which are in areas of social exclusion in the periphery of the city, while the other is located in a downtown district made up of the middle class. Participants in this study who were abstinent from the first weeks after treatment read and signed an informed consent form about the research that was going to be carried out, and voluntarily decided to participate.

Data collection was carried out in a health center consultation by two psychologists. The procedure consisted of administering a battery of the instruments described above to each patient individually. The duration was about 25 to 30 min. The batteries were administered when the patients entered treatment with the psychologist at the drug dependency unit.

### 2.4. Analytical Procedure

The analysis focused on the differences in means between the different groups of substances (alcohol, cannabis, cocaine, heroin and benzodiazepines) for the scores obtained in the variables of depression and cognitive impairment.

Pearson correlations were carried out on the different variables. Firstly, between the sociodemographic variables with the Beck inventory and the MoCA inventory of the present sample to find the existence of some type of relationship between age, age of onset of consumption and duration of consumption with depression and cognitive impairment. Secondly, among the sociodemographic variables that made up the initial questionnaire such as age, the age of onset of consumption and the duration of consumption in the sample to analyze the existence of some type of relationship in the sample of consumers of psychoactive substances. Thirdly, between the cognitive deterioration and sociodemographic variables according to each of the substances consumed to know whether cognitive impairment had a relationship with age, the age of onset of consumption and consumption time depending on the substance consumed.

Univariate analyses were also performed by multiple comparisons with the variables MoCA inventory and Beck inventory to analyze relationships between cognitive impairment or not and the four categories of the variable depression. Finally, all analyses were carried out with SPSS.

## 3. Results

### 3.1. Relationship between Sociodemographic Variables with Other Study Variables: Type of Substance, Depression Level and Cognitive Impairment

Regarding the sociodemographic variables, Pearson correlation analyses were performed to see if there was a relationship between current age, time of consumption and age of onset of consumption. A statistically significant positive correlation was found between the current age and the time of consumption (r = 0.81; *p* < 0.01). Moreover, a statistically significant positive correlation between the current age and the age of onset of consumption was found (r = 0.27; *p* < 0.01). Finally, there was a statistically significant negative correlation between the consumption time and age of onset of consumption (r = −0.35, *p* < 0.01). Pearson correlation analyses were performed to check if there was a relationship between the scores obtained in the Beck inventory (depression) and in the MoCA inventory (cognitive impairment) together with the sociodemographic variables (consumption time, age of onset of consumption and current age). The age of onset of consumption did not show any significant correlation with depression or cognitive impairment. A statistically significant negative correlation was found between the cognitive impairment variable with the current age (r = −0.22; *p* < 0.01) and time of consumption (r = −0.18; *p* < 0.05). The level of depression was not correlated with age and time of consumption.

### 3.2. Differences between Types of Substance and Depression Level or Cognitive Impairment

Univariate analysis was performed through multiple comparisons to evaluate whether there was a statistical difference between the scores in the Beck inventory (depression) and the different types of substances (cannabis, cocaine, heroin, alcohol and benzodiazepines). There was no significant difference found between the different consumer groups (cannabis, cocaine, heroin, alcohol and benzodiazepines) and the level of depression perceived (*F*(4,248) = 0.71, *p* = 0.59, eta2 = 0.01, potency = 0.23); so, post hoc analyses did not show any significant differences. Table 1 shows the mean scores in Beck’s questionnaire, standard deviations and percentage of consumers of psychoactive substances depending on the types of depression.

**Figure 1:**
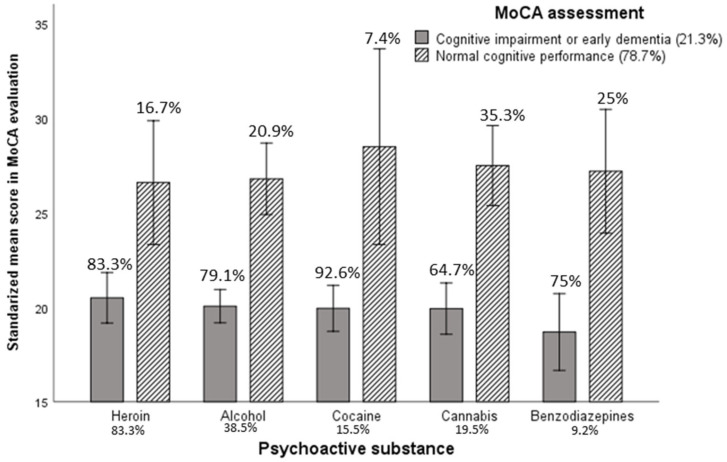
Mean scores of consumers of psychoactive substances in cognitive impairment classification across MoCA scores (Montreal Cognitive Assessment). Note: Scores range from 0 to 30: scores ≥ 26 indicate normal cognitive performance, while scores < 26 indicate mild cognitive impairment or early dementia.

Univariate analysis was performed to evaluate if there was any statistical difference between the scores in the MoCA inventory (cognitive impairment) and the different types of substances (cannabis, cocaine, heroin, alcohol and benzodiazepines). No significant differences were found among the different consumer groups (cannabis, cocaine, heroin, alcohol and benzodiazepines) and the cognitive deterioration registered (*F*(4,169) = 1.93; *p* = 0.11, eta2 = 0.04, potency = 0.57). However, post hoc analysis showed significant differences in the cognitive deterioration between the subjects consuming cannabis and those consuming cocaine (t = −0.28, *p* < 0.01), and marginal differences between cannabis and heroin, and between alcohol and cocaine (t = −0.18, *p* < 0.07; t = −0.28, *p* < 0.10, respectively). Figure 1 shows the scores in the MoCA assessment (Montreal Cognitive Assessment) across the cognitive impairment classification and the type of substance. When considering the whole sample, the worst score in cognitive impairment was from cocaine consumers (M = 20.70, SD = 0.90), followed by benzodiazepine (M = 20.94, SD = 1.17), alcohol (M = 21.24, SD = 0.57), heroin (M = 21.97, SD = 0.86) and cannabis (M = 22.24, SD = 0.81) consumers. Only 37 subjects were classified with normal cognitive performance. From those with cognitive impairment or early dementia, the worst score was from benzodiazepines consumers, followed by cannabis, alcohol, cocaine and heroin consumers.

**Figure 2:**
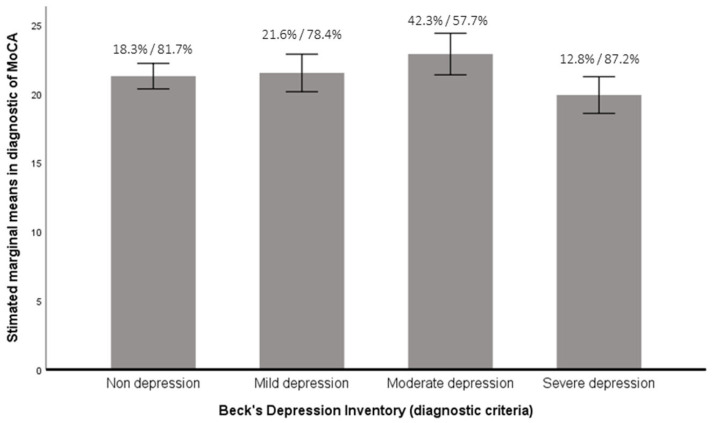
Mean differences in the level of depression experienced considering the score in cognitive impairment across MoCA (Montreal Cognitive Assessment, scores under 26 are considered as cognitive impairment). The percentages on each column indicate the distribution according to the diagnostic criteria in “normal cognitive performance” versus “cognitive impairment or early dementia”.

Finally, univariate analyses were performed with the variables of Beck’s inventory (depression) and the MoCA inventory (cognitive impairment) to analyze relationships between the four categories of the depression variable (non-depression, mild, moderate or severe) and cognitive impairment (in comparison with the MoCA score and the diagnostic criteria—with cognitive impairment or not). Univariate analyses were significant in both cases (*F*(3,169) = 3.12, *p* < 0.05, eta2 = 0.05, potency = 0.72; *F*(3,169) = 3.05, *p* < 0.05, eta2 = 0.05, potency = 0.71, respectively). It was found that there was a statistically significant difference between the moderate depression variable and the MoCA score inventory variable compared with the non-depression and severe depression variable (t = 2.39; *p* < 0.05; t = 3.49; *p* < 0.01, respectively). We also found similar results when the MoCA diagnostic criteria were utilized; the moderate depression variable had a statistically significant difference with the MoCA diagnostic criteria compared to the non-depression, mild, and severe depression variables (t = −0.24; *p* < 0.01; t = −0.21; *p* < 0.05, and t = −0.30; *p* < 0.001, respectively). See Figure 2 to observe the mean differences in depression with regard to cognitive impairment.

## 4. Discussion

The results of the present study show that there are no significant differences in the levels of depression among the subjects according to the substance consumed. Subjects with a more severe diagnosis of depression were the consumers of benzodiazepines, followed by consumers of alcohol, heroin, cocaine and cannabis, in different percentages. According to the literature found on the one hand, high depressive symptomatology without the diagnosis of depression is observed in alcohol and cannabis users, although on the other hand, there are many subjects diagnosed with depression, not only with the symptomatology, in the case of alcohol [17,18,19].

According to research by Rojo-Mota et al. [11], no significant differences were observed in the levels of cognitive deterioration among the subjects depending on the substance consumed. However, here, a significant difference was found between cannabis and alcohol consumers, which would indicate that people who consume alcohol obtain a lower score in the MoCA inventory, showing greater cognitive deterioration than people who use cannabis, or similarly, that cannabis users have less cognitive impairment than alcohol users. However, it is necessary to highlight that these results obtained indicate that cognitive impairment was detected in 78.7% of participants compared to non-cognitive impairment in 21.3% of the sample analyzed. It should also be noted that cocaine users have the worst level of cognitive impairment and cannabis users have the best cognitive functioning, similar to results reported by Rojo-Mota et al. [11] and Bruijnen et al. [8].

Regarding the sociodemographic variables, a statistically significant relationship was found between cognitive deterioration and the variables of the subject’s age and the time of consumption. This would indicate that a lower score obtained in the MoCA inventory, which shows a greater cognitive deterioration, is linked to a greater current age and time of consumption found in the subjects. Therefore, consumers of toxic substances that have greater cognitive deterioration are older and have spent more time consuming. Contrary to other studies [11], where no statistically significant relationships were found between the cognitive deterioration of consumers and the time of consumption, in the present study, a statistically significant relationship was also found between the sociodemographic variable of the subject’s current age, time of consumption and age of onset. This indicates that people with a more advanced age have been consuming psychoactive substances for longer and began to consume at later ages. Likewise, a statistically significant relationship was observed between the time of consumption and the age of onset, which would mean that consumers who have been spending less time consuming these types of substances began to consume them at a later age.

In the statistically significant results found concerning the relationship between cognitive deterioration and the different sociodemographic variables depending on the substance consumed, it was found that the heroin, alcohol and benzodiazepine users who obtained a lower score in the MoCA inventory, which would mean greater cognitive deterioration, were older. This emphasizes that consumers of benzodiazepines with a lower score in the MoCA inventory, that is, with greater cognitive deterioration, began their consumption at a later age. Finally, with regard to the cocaine and cannabis users, no statistically significant relationships were found between cognitive deterioration and the current age of the subjects. A statistically significant relationship was also found in the consumers of alcohol, cocaine and benzodiazepines between the variables of current age and age of onset of consumption. These results showed that the consumers of these three substances with greater age began the consumption of these at a later age.

With regard to the variable consumption time, three statistically significant relationships were found with the MoCA inventory variables, current age and age of onset of consumption depending on the substance consumed. Firstly, regarding the cognitive impairment and time of consumption variables, the findings demonstrated that, in heroin and alcohol users, subjects who had higher scores in the MoCA inventory, with less cognitive decline, had been consuming for less time. Secondly, the variables of consumption time and current age showed a statistically significant relationship in the users of heroin, alcohol, cocaine and cannabis. As such, consumers that have consumed for a greater period of time are older. Thirdly, with regard to the variables of time of consumption and age of onset, statistically significant relationships were found in the consumers of heroin, alcohol, cocaine, cannabis and benzodiazepines.

As for the substances analyzed, for alcohol consumers, the age of onset of consumption was at 16.65 years, which is comparable with the results of other studies that reported an age of 16.8 years. It was also found that the duration of consumption was 29.52 years, which was greater than the 14.9 reported in other studies [32].

Finally, in relation to the relationship between cognitive impairment and depression in consumers of these types of substances, as in other recent studies [33], a series of statistically significant relationships were found. On the one hand, it was observed that consumers who did not have depression scored lower in the MoCA inventory than people who had moderate depression. This shows that consumers without a diagnosis of depression have greater cognitive deterioration than those who have moderate depression. Or, similarly, subjects with moderate depression have lower cognitive impairment than those who do not have a diagnosis of depression. On the other hand, consumers with moderate depression scored higher in the MoCA inventory, that is, they had lower cognitive deterioration than people with severe depression. This, in turn, means that consumers with severe depression have greater cognitive decline than those diagnosed with moderate depression.

One of the limitations of this study is its exploratory design. It was intended to analyze the relationship between the type of consumption with depression and existing cognitive impairment. Although the age of onset of use and years of substance use were included in the present investigation, other studies have also assessed the residual harm of drug use [34,35,36]. Future research could use a longitudinal design to assess the impact of sustained use over the years on depression and cognitive impairment. In addition, a longitudinal design could shed light on the directionality and causality of the relationships studied. Another limitation comes from the classification of the sample according to the main substance of use; however, most drug users are polydrug users or have been combining the type of substance over the years. In future studies, it would be interesting to analyze whether different combinations of drug use lead to different symptoms of depression or cognitive impairment.

## 5. Conclusions

This study aimed to show the level of cognitive deterioration and depression in consumers of psychoactive substances, as well as the relationship that exists between these two variables and age, age of onset of consumption and duration of consumption. To conclude, it was found that a high percentage of consumers of psychoactive substances have alterations in cognitive level and in depression; this result is useful for future intervention programs. Given the high prevalence of depression and cognitive impairments in those who consume psychoactive substances, early psychological treatment is recommended to avoid greater cognitive and emotional impact. Therefore, the incorporation of psychological treatment in primary care would be effective for the improvement of psychoactive substance abusers who report high levels of anxiety and depression. Similarly, it seems necessary to try to avoid pharmacological treatments sustained over time to avoid the sequelae of cognitive impairment.

## Figures and Tables

**Table 1:** Percentage of consumers of psychoactive substances depending on the types of depression.

	BDI Score	Type of Depression			
Substance	Mean (SD)	No Depression	Mild Depression	Moderate Depression	Severe Depression
Heroin	17.76 (2.25)	33.3	24.2	24.2	18.2
Alcohol	18.02 (1.24)	33.0	23.9	12.8	30.3
Cocaine	16.80 (2.07)	38.5	25.6	7.7	28.2
Cannabis	15.39 (1.81)	43.1	21.6	15.7	19.6
Benzodiazepines	20.57 (2.82)	28.6	19.0	19.0	33.3

## Data Availability

Not applicable.
